# Supplementary material for: Antibacterial efficacy and possible mechanism of action of 2-hydroxyisocaproic acid (HICA)
Source: PLoS One. 2022 Apr 1;17(4):e0266406. doi: 10.1371/journal.pone.0266406 (PMC8975099; doi:10.1371/journal.pone.0266406)
Supplement: S1 Table — (DOCX) [file pone.0266406.s001.docx]

S1Table. Bacterial growth inhibition by different HICA concentrations

| **Microorganisms** | **HICA concentration (mg/mL)** | | | | | | | |
| --- | --- | --- | --- | --- | --- | --- | --- | --- |
|  | **0.25** | **0.5** | **1** | **2** | **4** | **8** | **16** | **32** |
| *Shewanella putrefaciens* SM26 (meat isolate) | G/C | NG/C | NG/NC | NG/NC | NG/NC | NG/NC | NG/NC | NG/NC |
| *Serratia proteamaculans* ENT68 (meat isolate) | G/C | G/C | NG/C | NG/NC | NG/NC | NG/NC | NG/NC | NG/NC |
| *Escherichia coli* O157:H7 NCTC12900 | G/C | G/C | NG/C | NG/NC | NG/NC | NG/NC | NG/NC | NG/NC |
| *Escherichia coli* AGR3789 (soil isolate) | G/C | G/C | NG/C | NG/NC | NG/NC | NG/NC | NG/NC | NG/NC |
| *Pseudomonas aeruginosa* ATCC25668 | G/C | G/C | NG/C | NG/NC | NG/NC | NG/NC | NG/NC | NG/NC |
| *Pseudomonas aeruginosa* NZRM4034 | G/C | G/C | NG/C | NGNC | NG/NC | NG/NC | NG/NC | NG/NC |
| *Pseudomonas lundensis* F2MCUH2 (environmental isolate) | G/C | G/C | NG/C | NG/NC | NG/NC | NG/NC | NG/NC | NG/NC |
| *Pseudomonas fragi* F1NBUH38 (environmental isolate) | G/C | G/C | NG/C | NG/NC | NG/NC | NG/NC | NG/NC | NG/NC |
| *Staphylococcus aureus* NZRM917 | G/C | G/C | NG/C | NG/NC | NG/NC | NG/NC | NG/NC | NG/NC |
| *Bacillus mycoides* ATCC6462 | G/C | G/C | NG/NC | NG/NC | NG/NC | NG/NC | NG/NC | NG/NC |
| *Bacillus subtilis* F2MCUH1 (environmental isolate) | G/C | G/C | NG/C | NG/C | NG/NC | NG/NC | NG/NC | NG/NC |
| *Paenibacillus odorifer* F1OSP28 (environmental isolate) | G/C | G/C | NG/C | NG/C | NG/NC | NG/NC | NG/NC | NG/NC |
| *Bacillus cereus* NZRM5 | G/C | G/C | NG/C | NG/C | NG/C | NG/C | NG/C | NG/NC |
| *Bacillus cereus* M4 (milk isolate) | G/C | G/C | NG/C | NG/C | NG/C | NG/C | NG/C | NG/NC |

G – Growth (OD change observed after 24 h incubation)

NG – No Growth (no OD change observed after 24 h incubation)

C – Colonies observed on SBA plates

NC – No Colonies observed on SBA plates

(Results were obtained after testing three replicates and G and NG were used to determine MIC and C and NC were used to determine MBC)
