# Supplementary material for: Antibacterial efficacy and possible mechanism of action of 2-hydroxyisocaproic acid (HICA)
Source: PLoS One. 2022 Apr 1;17(4):e0266406. doi: 10.1371/journal.pone.0266406 (PMC8975099; doi:10.1371/journal.pone.0266406)
Supplement: Attachment — Submitted filename: Response to reviewers 2.docx [file pone.0266406.s003.docx]

We thank reviewers for reviewing our work.

1. The lack of pH controls needs to be at least discussed. Ideally the experiments would be repeated with them, but an insightful discussion may suffice. It is well known that some microbes tolerate low pH poorly whereas others benefit from it. It would be important to know if the effects seen are actually due to the antimicrobial effect of HICA or the low pH (as low as 3 as stated in the methods).

This has been identified and discussed in the discussion segment. The lack of information on clarifying the relationship between low pH of HICA and its antimicrobial activities in the current work and the need for future studies to fill that knowledge have been identified and discussed. Please see lines 547 to 562

2. I note that the word antimicrobial has been changed to antibacterial throughout. I presume this is due to comments made by the reviewers. The change is correct in some instances but in others not. HICA does have antifungal activity (see reference below) and when described more broadly antimicrobial activity is more appropriate than antibacterial activity. The authors need to go through each of those changes and check what exactly they mean and which of the two words is more appropriate.

We thoroughly checked the manuscript and changed the terminology accordingly.
